# Successful transarterial embolization with cellulose porous beads for occipital haemangioma in an infant with Kasabach-Merritt syndrome

**DOI:** 10.1259/bjrcr.20170004

**Published:** 2017-05-10

**Authors:** Zaw Aung Khant, Toshinori Hirai, Osamu Ikeda, Eiji Furukoji, Yoshihito Kadota, Minako Azuma, Norihiro Shinkawa, Keiji Kitatani, Yoichi Mizutani, Kimihiko Endo, Yasuyuki Yamashita

**Affiliations:** ^1^Department of Radiology, Faculty of Medicine, University of Miyazaki, Miyazaki, Japan; ^2^Department of Diagnostic Radiology, Graduate School of Medical Sciences, Kumamoto University, Kumamoto, Japan

## Abstract

We report a 3-month-old boy with Kasabach-Merritt Syndrome (KMS) with an occipital haemangioma who underwent successful transarterial embolization (TAE) with cellulose porous beads (CPBs). As his response to steroids and coil embolization was inadequate, we performed TAE with CPBs, carefully preventing their migration via dangerous anastomoses. The tumour blush decreased, there were no complications, all coagulation tests were immediately normalized and the tumor size decreased gradually. TAE with CPBs is useful for the treatment of KMS.

## Background

KMS was first reported by Kasabach, a radiologist, and Merritt, a pediatrician, in 1940.^1^ About 80% of infants present within their first year; the reported mortality rate ranges from 10 to 37%.^2^ KMS treatment is aimed at reducing the tumor size, controlling coagulopathy and preventing significant mortality and morbidity. While there are no consensus guidelines for its treatment, good therapeutic effects were obtained with systemic corticosteroids, propranolol, interferon, chemotherapy, radiotherapy, transarterial embolization (TAE) and surgery.^2–8^ Wang et al.^3^ reported that while steroid therapy was effective in 35.3% of their patients, the relapse rate was 50%. Adverse effects argue against the prolonged administration of steroids. Although some patients respond to interferon when steroid treatment fails, life-threatening adverse events and its high cost prevent its wider use.^4^ Radiotherapy immediately increases the platelet count, but the potential for growth retardation and radiation-induced malignancy is a significant problem^5^ Surgical intervention may be the most definitive treatment option but may be impossible owing to the size and invasive nature of most tumours at the time of diagnosis.^6^ Embolization seems to be safe and effective; it yields both haematological cure and involution although complications may occur.^9^ Here we report successful TAE with CPBs for an occipital hemangioma in an infant with KMS.

## Case report

Prior written informed consent for the use of the embolic materials was obtained from the 3-month-old infant’s legal representatives. He harboured a semi-spherical tumour in the right occipital region, and coagulation tests at birth were abnormal; a diagnosis of KMS with occipital haemangioma was made. On day 0, he was started on prednisolone followed by propranolol and he received platelet transfusions due to a low platelet count. On day 36 he underwent transarterial coil embolization of the haemangioma feeders at another hospital. His platelet count increased transiently (50–70 × 103 μl^−1^) without further transfusions; however, thrombocytopenia worsened again when the prednisolone dose was tapered and at the age of 3 months he was referred to our hospital for further management.

**Figure 1.**
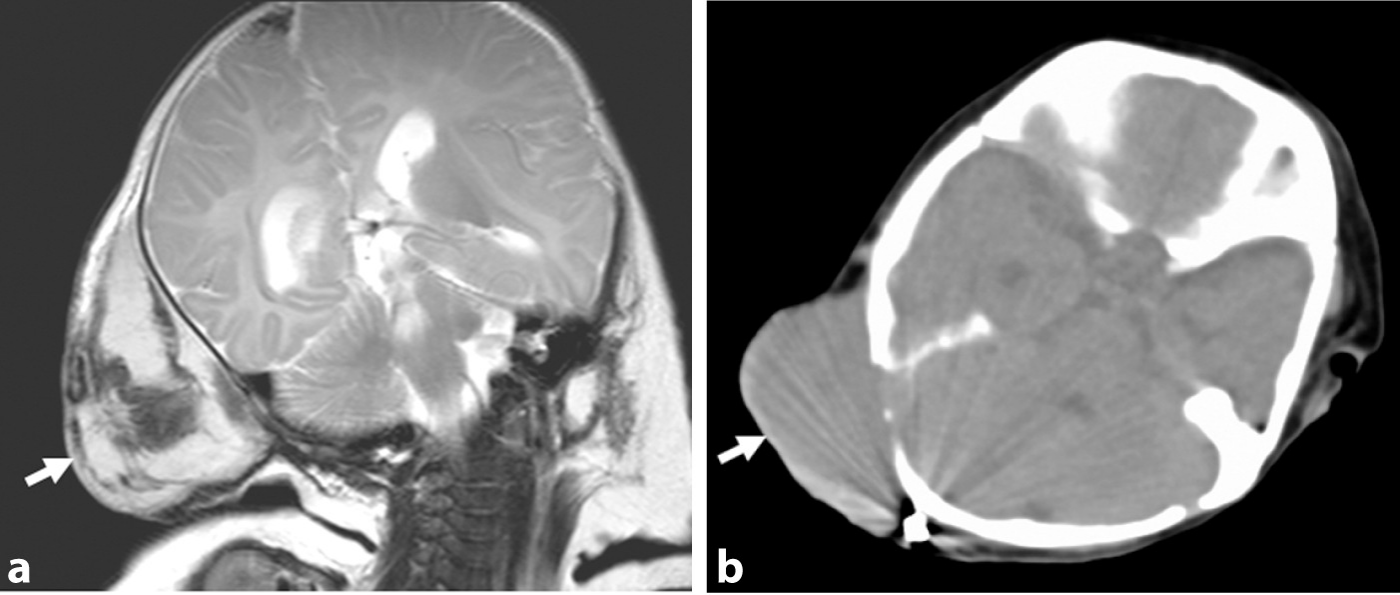
MRI and CT images obtained at the age of 3 months. (a) *T*_2_-weighted MRI showing a large mass with mixed intensity in the right occipital region (arrow). (b) CT scan showing a large mass with bony structure involvement in the right occipital region (arrow).

At the time of admission, his general condition and limb movements were normal. A soft haemangioma (9 × 6 cm) with a purple surface was present in the right occipital region. The results of laboratory tests were: haemoglobin 11.0 dl^−1^, platelet count 107 × 103 μl^−1^, fibrin degradation product 19.0 μg ml^−1^ and D-dimer 9.7 μg ml^−1^. CT and MRI scans showed a large mass in the right occipital region with bony structure involvement (Figure 1).

**Figure 2.**
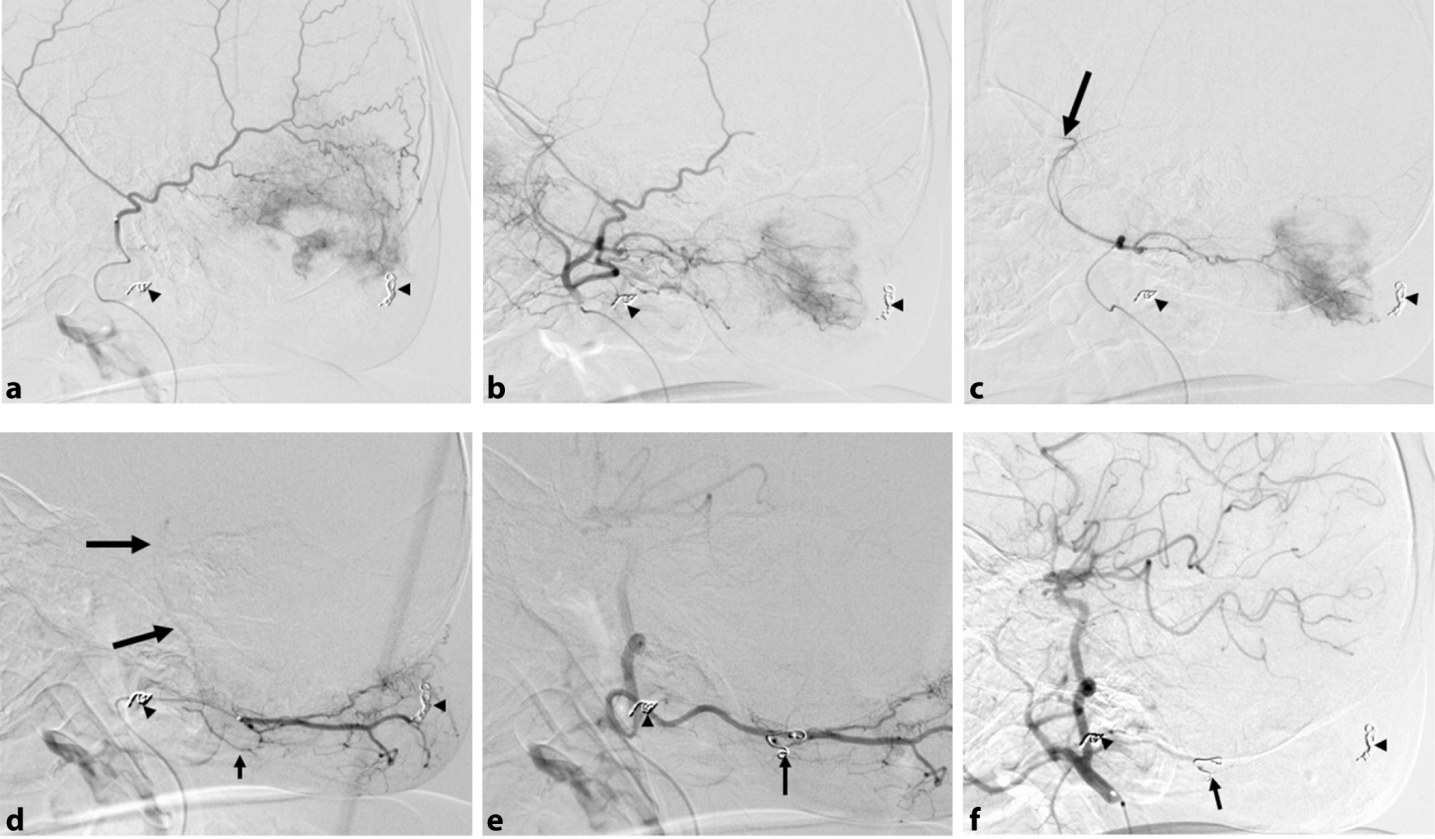
Digital subtraction angiograms (DSAs) performed during embolization. Small arrowheads indicate previously placed embolization coils. (a) Superselective DSA from the right superficial temporal artery shows tumour blush in the occipital region. (b) DSA from the external carotid artery showing a marked post-embolization decrease in tumour blush. (c) Superselective DSA from the right middle meningeal artery shows the lacrimal artery (arrow) and a tumour blush. After advancing the microcatheter tip distally to the orifice of the lacrimal artery to prevent the migration of embolic materials into the orbital structures, the feeding artery was embolized. (d) Superselective DSA from the right occipital artery reveals a tumour blush and the vertebrobasilar artery (large arrows) via the C1 collateral (small arrow). (e) The anastomosis was embolized before coil embolization. (f) DSA from the external carotid artery after final embolization shows a marked decrease in the tumour blush. The arrow points to the coil used to embolize the C1 collateral.

With the patient under general anaesthesia, we percutaneously inserted a 4-French sheath into the left femoral artery under ultrasound guidance. A 4-French JB1 catheter was introduced into the right common carotid artery. Digital subtraction angiography (DSA) of the right carotid artery showed a hypervascular lesion fed by the right superficial temporal, middle meningeal and occipital arteries. Using a 4-French diagnostic catheter as a guiding catheter, a 2.5-French Renegade microcatheter (Boston Scientific Corp.) was selectively positioned at the distal portion of the right superficial temporal artery (Figure 2a) and CPBs (230 μm) suspended in contrast medium were slowly injected until the tumour blush disappeared on DSA (Figure 2b). Then the right middle meningeal artery was similarly embolized after the tip of the microcatheter was advanced distally to the orifice of the lacrimal artery to prevent the migration of CPBs into the orbital structures (Figure 2c). As DSA of the right occipital artery revealed not only tumour blush but also the vertebrobasilar artery (Figure 2d), we used interlocking detachable coils to embolize the dangerous anastomosis of the C1 collateral from the occipital artery (Figure 2e). Subsequently the occipital artery was safely embolized with CPBs. Embolization of the three feeders was successful and there was a marked decrease in the tumour blush (Figure 2f). Although 2 h manual compression at the puncture site of the sheath was required, there were no complications associated with the procedure.

**Figure 3.**
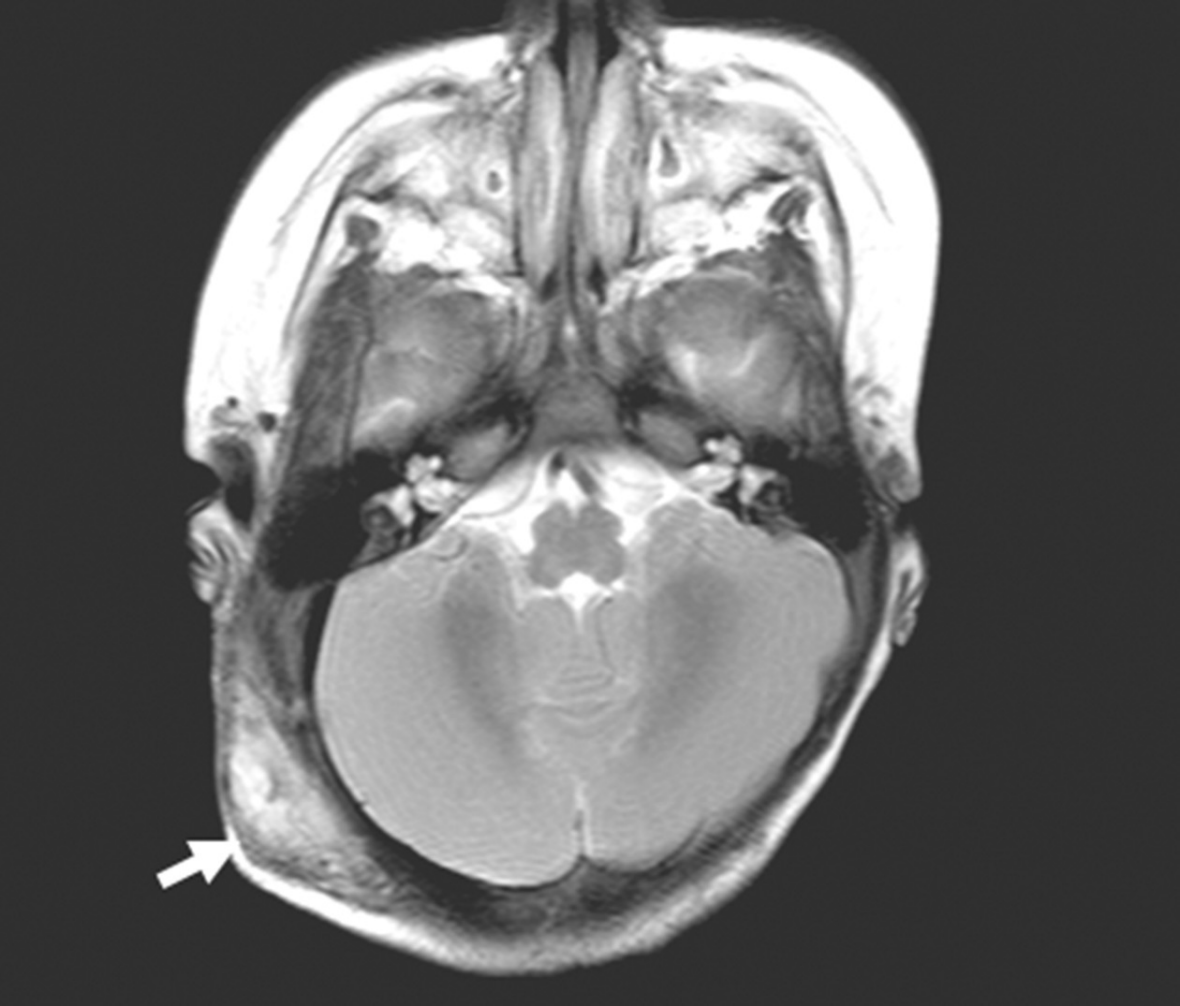
The *T*_2_ weighted MRI obtained 6 months after embolization shows a significant decrease in the tumour size (arrow).

As all post-embolization coagulation tests were immediately normalized, the administration of corticosteroid and propranolol was stopped. The tumour size decreased gradually and the tumour was remarkably smaller 6 months after embolization (Figure 3). It was not palpable when our patient was 2 years of age.

## Discussion

This infant with KMS with an occipital haemangioma had failed to respond to drug therapy and coil embolization. With transarterial CPBs embolization we subsequently obtained a good outcome. Although the CPB embolization of meningiomas, dural arteriovenous fistulae, paragangliomas and spinal arteriovenous malformations has been reported,^10–12^ to our knowledge, ours is the first documentation of successful transarterial embolization with CPBs to address haemangioma with KMS.

In patients who fail to respond to conservative medical treatment, TAE represents an effective therapeutic option. The procedure involves the use of embolic materials such as coils, polyvinyl alcohol (PVA) particles and onyx to address haemangiomas presenting with KMS.^7,8,13^ Coils facilitate the abrupt, proximal occlusion of the feeding vessel. However, these tumours can be fed by multiple vessels supplying nearby territories.^7^ PVA particles are effective in the acute setting but recanalization may occur as they tend to “wash away” with time^7^ The CPBs we used facilitate permanent occlusion in the clinical setting. They can be injected easily through microcatheters, they travel to distal sites, remain in suspension for prolonged periods without clumping and they produce more complete and permanent embolization without eliciting inflammatory changes.^10–12^ They are different from other embolic materials in that they cannot be absorbed, their specific gravity is similar to that of whole blood, they are round, uniform and small in size, their surface is smooth, and as they are positively charged, the particles repel each other.^10–12^

Intraoperatively, we detected a dangerous ophthalmic branch and an anastomosis to the vertebrobasilar artery and we were able to prevent the migration of CPBs into these vessels. Our experience alerts to the possible presence of dangerous branches in patients with head and neck hemangioma presenting with KMS. Therefore, embolization must be performed very carefully to prevent serious neurologic complications, especially when small or liquid embolic materials are used.

## Conclusions

We report an infant with an occipital hemangioma presenting with KMS whose treatment with drugs and coil embolization had failed. Subsequent embolization with CPBs was successful, his coagulopathy improved immediately and the size of his hemangioma decreased significantly. Care must be taken to rule out anastomoses that would allow the migration of small and liquid embolic materials and the elicitation of neurologic sequelae.

## Learning points

1. TAE with CPBs is useful for the treatment of KMS.
2. In patients who fail to respond to conservative medical treatment, TAE with CPBs is an effective therapeutic option.
3. Care must be taken to rule out anastomoses that would allow the migration of small and liquid embolic materials and the elicitation of neurologic sequelae.

## Consent

Written informed consent for the case to be published (including images, case history and data) was obtained from the patient's parents. 
